# Influence of Solvent and Organophilic Clay Content on the Properties of EVA Nanocomposite Films

**DOI:** 10.1021/acsomega.5c12672

**Published:** 2026-04-02

**Authors:** Adeilson de Oliveira Souza, Anthunes Íkaro de Araújo, José César Augusto de Queiroz, Michelle Cequeira Feitor, Thercio Henrique de Carvalho Costa, Kaline Melo de Souto Viana, Maxwell Santana Libório, Amanda Melissa Damião Leite

**Affiliations:** † School of Science and Technology, 28123 Federal University of Rio Grande do Norte, Natal, Rio Grande do Norte 59075-000, Brazil; ‡ Postgraduate Materials Science and Engineering Program, Federal University of Rio Grande do Norte, 59075-000 Natal, Rio Grande do Norte, Brazil; § Postgraduate Mechanical Engineering, Federal University of Rio Grande do Norte, Natal, Rio Grande do Norte 59075-000, Brazil; ∥ Post-graduation of Aerospace Engineering, Federal University of Rio Grande do Norte, Natal, Rio Grande do Norte 59075-000, Brazil

## Abstract

This study investigates the combined influence of solvent type and organophilic clay (Cloisite 20A) content on the properties of EVA nanocomposite films with a view to improving mechanical and barrier performance. EVA (vinyl acetate) films were obtained by solvent evaporation, using chloroform (CHCl_3_) or tetrahydrofuran (THF) as the solvent, and with 0, 3, and 5% clay as reinforcement. XRD, FTIR, SEM, TGA, DSC, contact angle, water vapor transmissibility, and tensile testing were used to characterize the samples. Structural and morphological analyses indicated good lamella dispersion and heterogeneous morphology throughout the thickness. The incorporation of clay increased thermal stability (lower mass loss up to ∼ 300 °C) and low-temperature melting enthalpy, suggesting a higher crystalline fraction and greater rigidity. The Young’s modulus of the ETC3% film (THF + 3% clay) was more than twice that of ETC0 %. THF-based films showed lower elongation at break, while those produced with chloroform were more ductile. Clay increased the contact angle and reduced water vapor permeability; the ECHC3 % film showed a ∼37% reduction, and the ETC3 % film showed a ∼15% reduction compared to the respective pure films, highlighting the relevance of the solvent as a design parameter in EVA/organoclays films.

## Graphical abstract

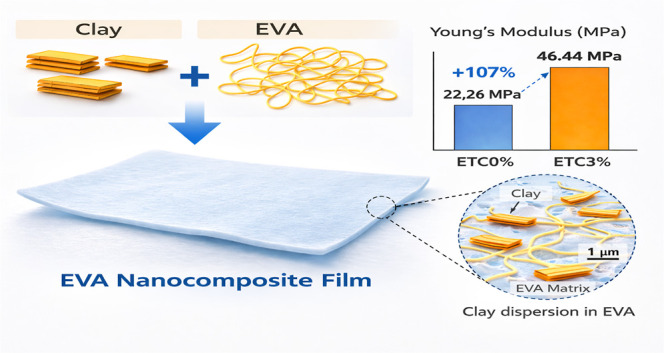

## Introduction

Currently, the development of multifunctional polymeric materials has been the subject of ongoing research in materials science and engineering, due to their combination of characteristics, such as lightweight, flexibility, and specific barrier properties.^1,2^ Among the widely researched polymers, ethylene vinyl acetate (EVA) stands out for its versatility of applications, satisfactory processing performance, and ability to adapt to different conditions of use.^2^

This material has a semicrystalline structure resulting from the copolymerization of ethylene and vinyl acetate, offering a balanced combination of elasticity and rigidity and making it a unique candidate for applications ranging from encapsulation of photovoltaic modules to packaging films and functional membranes.^1–3^ However, EVA has limitations in specific properties, such as high permeability to gases and water vapor, as well as low thermal stability, which limit its application to systems that require high barrier efficiency or resistance to extreme environmental conditions.^4,5^

To overcome these limitations, recent research has developed studies on the modification of polymers with lamellar particles, which have emerged as an efficient strategy that exploits the reinforcing effect and increases the tortuosity of molecular diffusion paths.^6^ In this context, montmorillonite clays modified by organic surfactants, generically known as organoclays, stand out. This material, well established in the literature, has been widely used in the production of polymer nanocomposites due to its high aspect ratio and ability to generate nanometric barriers to gas diffusion.^7,8^ When uniformly dispersed, these lamellae act as physical barriers to the migration of gas and vapor molecules, in addition to inducing significant changes in the morphology, crystallinity, and thermal and mechanical properties of the host polymer/polymer matrix.^9,10^ This synthesis methodology, which combines simplicity of processing and performance gains, has shown promise for both sustainable packaging applications and functional coatings.^11,12^

Morita et al.^2^ observed that the optimized dispersion of modified clays in EVA led to a significant increase in thermal stability and a change in the composite’s shear viscosity, indicating strong interfacial interactions between EVA and the clay. Similarly, Wilson et al.^10^ found that moderate clay contents (∼3%) are sufficient to improve the gas barrier without compromising mechanical integrity, provided that the lamellae are homogeneously distributed in the polymer matrix. Several review articles in the literature point out that this direct correlation between morphology and properties is one of the pillars of polymer nanocomposite engineering.^2,10,13–15^ Tsurko et al.^16^ report that, in many cases, the choice of solvent is made empirically, without a clear understanding of the resulting microstructural effects. However, the combined influence of the solvent and clay content on the morphological and functional behavior of the EVA/Cloisite system is important and relatively scarce in the literature.

Although a significant number of studies have been devoted to EVA-based nanocomposites reinforced with organophilic clays, most of the available literature has focused on the effects of clay type, surface modification, compatibilizer addition, and filler content on the structural, thermal, and mechanical properties of the materials. In these studies, the solvent is generally treated as a secondary processing variable, being primarily used as a medium for polymer dissolution and film formation, with limited discussion of its physicochemical influence on the microstructural evolution of the nanocomposite.^9,10,13^

More recent investigations indicate that solvent-related parameters, such as polarity, solubility affinity with the polymer matrix, and evaporation kinetics, can significantly affect polymer chain organization, the degree of clay intercalation or exfoliation, and the development of crystalline and amorphous domains in polymer/clay systems.^16–18^ However, in EVA-based nanocomposites, these effects are typically addressed indirectly or under specific processing conditions, without a systematic comparison between different solvents while keeping other synthesis parameters constant.^2,10,11^ As a result, the role of solvent selection as an active processing parameter capable of governing the final morphology and structure–property relationships remains insufficiently explored in the literature.

In this context, the present study provides a comparative analysis of EVA/Cloisite 20A nanocomposite films prepared by solvent evaporation using chloroform and tetrahydrofuran, combined with controlled clay contents (3 and 5 wt %). By correlating solvent-dependent morphological features with thermal, mechanical, wettability, and water vapor barrier properties, this work contributes to clarifying how solvent selection modulates nanocomposite performance beyond the isolated effect of clay loading.

## Materials and Methods

Ethylene vinyl acetate copolymer (EVA) containing 19% vinyl, with a density of 0.940 g/cm^3^ and a melt flow index of 0.25 g/min, was used as the polymer matrix. Chloroform (CHCl_3_) and tetrahydrofuran (THF) solvents with densities of 1.48 g/cm^3^ and 0.88 g/cm^3^, respectively, were used to produce the films. Organophilic montmorillonite clay, a type of smectite clay in powder form with a particle size of less than 74 μM, was used to reinforce the polymer films.

The selection of chloroform and tetrahydrofuran (THF) as solvents was based on their documented ability to dissolve EVA effectively for solution casting processes and to form homogeneous polymer films after solvent evaporation. Previous work has used THF and chloroform for dissolving EVA in membrane and film fabrication studies, demonstrating their suitability for preparing polymer solutions with adequate solubility and controlled evaporation behavior.^19,20^ While other solvents like acetone and toluene are sometimes used in polymer processing, they can offer less favorable solvency or evaporation profiles for EVA under comparable processing conditions.^21^ In industrial and pharmaceutical film casting of EVA, chlorinated solvents such as chloroform are commonly reported due to their ability to solvate EVA and give defect-free films upon evaporation.^22^

The solvent evaporation method was used to produce EVA films and EVA/clay nanocomposites with 3% and 5% clay relative to the polymer mass. Initially, the solvent-clay solution was homogenized for 4 h, and then EVA was added to the solution. The films produced with and without reinforcement (clay) had a polymer/solvent mass ratio of 12/88 (12% EVA) and were subjected to mechanical agitation for 1 h at 60 °C. After agitation, the solution was spread on a glass plate and left to evaporate completely under ambient conditions for 2 h, with the solvent (CHCl3 or THF) removed. Table 1 presents the nomenclature for the samples, including the polymer (E), the solvent (CH or T), and the clay content (C0%, C3%, and C5%).

**Table 1:** Nomenclature of Polymer Films Produced by Evaporation

films (%)	solvent	clay (%)
ECHC0	CHCl_3_	0
ECHC3		3
ECHC5		5
ETC0	THF	0
ETC3		3
ETC5		5

The clay and films were analyzed by X-ray diffraction using a Shimadzu XRD-7000 diffractometer with Cu Kα radiation (1.5418 Å), 40 kV, and 30 mA, scanning from 2° to 30° with an angular step of 0.02°. The morphology of the films was analyzed by scanning electron microscopy using a Hitachi TM3000. Fourier transform infrared (FTIR) analysis was performed on a Bruker Vertex 70 instrument over 400–4000 cm^–1^, with a resolution of 4 cm^–1^ and 16 scans, using ATR. Thermogravimetry (TGA) was performed using a PerkinElmer THA Pyris-1 instrument. In this analysis, 5 to 10 mg of sample was placed in an aluminum capsule, which was heated from 30 to 600 °C under a synthetic gas flow of 20 mL/min and a heating rate of 10 °C/min. The wettability of the films was analyzed by measuring the contact angle using the sessile drop method with distilled water on the Phoenix-i equipment from surface electro optics (SEO). The differential scanning calorimetry (DSC) test was performed using TA Instruments (USA) DSC Q20 equipment. Five milligrams of each film were tested under a nitrogen gas flow of 50 mL/min, with heating at 10 °C/min from 30 to 150 °C. The water vapor transmissibility test was conducted in accordance with ASTM E96. Three samples of each synthesis condition (0%, 3%, and 5% clay) were used. Tensile tests were conducted in accordance with ASTM D882-12 to obtain the stress–strain curve for each film produced.

## Results and Discussion

The structural characterization of the nanocomposites and the evaluation of the degree of dispersion and intercalation of the organophilic clay (Cloisite 20A) in the polymer matrix (EVA) were performed by X-ray diffraction (XRD). The diffractograms obtained for the pure samples (clay, EVA, ECH0 %, and ETC0 %) and for the nanocomposites (ECHC3 %, ECHC5 %, ETC3 %, and ETC5 %) are shown in Figure 1.

**Figure 1:**
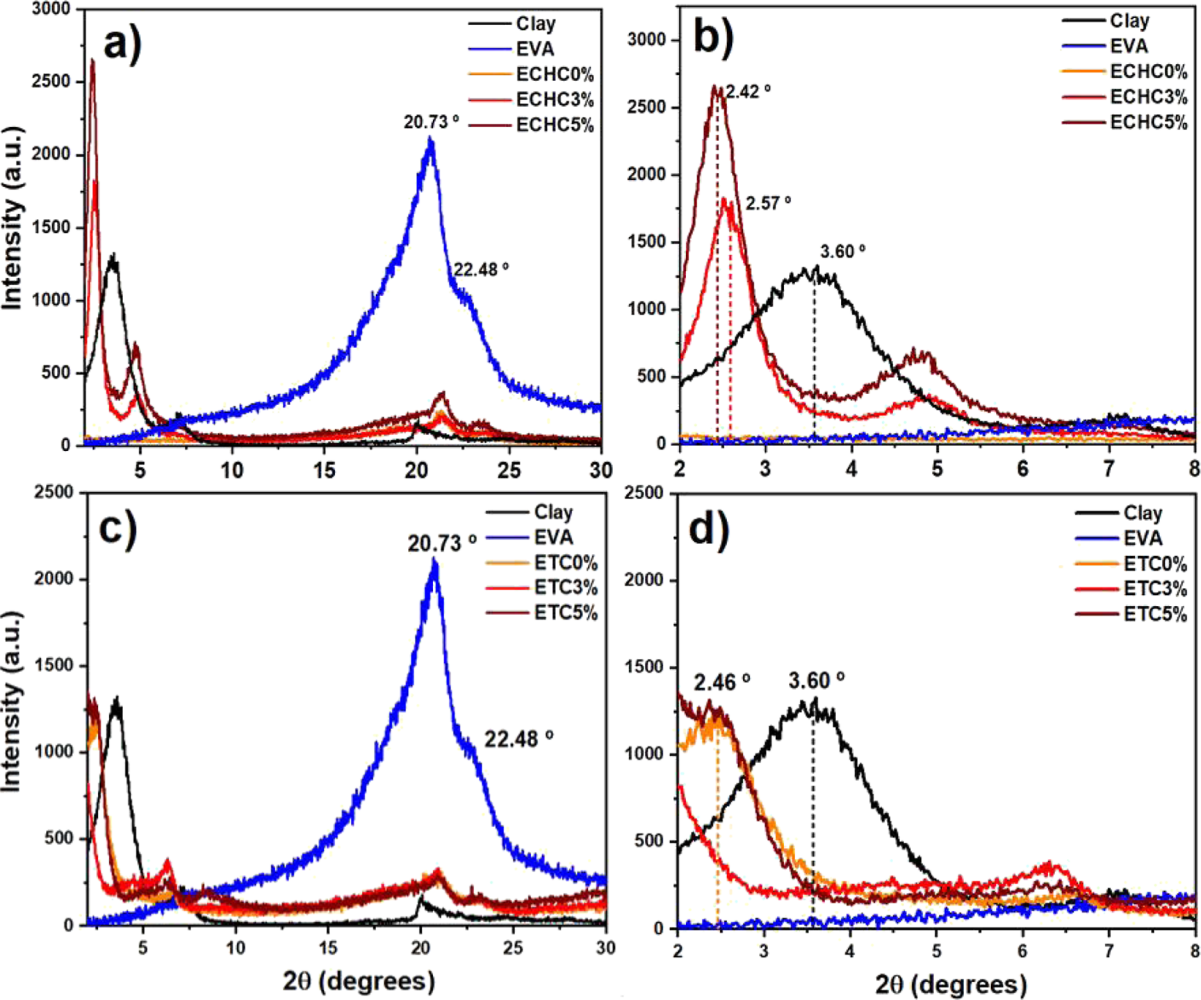
X-ray diffraction of clay, EVA, and EVA/Cloisite 20A films: (a) chloroform-based films (ECHC0 %, ECHC3 %, and ECHC5 %); (b) enlarged low-angle region of (a); (c) THF-based films (ETC0 %, ETC3 %, and ETC5 %); and (d) enlarged low-angle region of (c).

X-ray diffraction of clay, EVA, and polymer films with chloroform and THF as solvents.

The EVA sample presented a crystalline region with an intense peak at 20.73° and a shoulder at 22.48°.^23^ The films with and without clay showed no significant displacement of these peaks, but their intensities were significantly reduced, indicating an increase in the amorphous region. This may have been caused by greater polymer chain scattering upon dissolution in the solvent, resulting in less chain organization and, consequently, lower crystallinity in the films compared to EVA in pellet form.^17^

Cloisite 20A clay exhibited an intense diffraction peak at 3.60°, corresponding to an interplanar spacing of 24.5 Å. This value is typical of intercalated organophilic clays, and the shift observed in the samples synthesized with 3% and 5% clay indicates the penetration of polymeric macromolecules between the montmorillonite lamellae.^18,24,25^ In Figure 1a, a high-intensity peak is observed in the ECHC5 % sample, which can be attributed to a higher concentration of intercalated domains or to a more ordered lamellar stacking with increasing clay content.^26,27^

The unreinforced films (ECHC0 % and ETC0 %) showed a predominantly amorphous profile over the analyzed angular range, with a low-intensity peak around 23°. However, the ETC0 % sample also showed a semicrystalline peak at 2.46°.

Figure 2 shows electron microscope images of the cross-sectional profile of films solubilized in chloroform (Figure 2a–c) and THF (Figure 2d–f). Films without clay in their composition (Figure 2a,d) exhibit a dense, continuous microstructure with no interconnected porosity. According to García-Munoz,^27^ the upper region, marked by fracture lines resulting from the cut, indicates the occurrence of cleavage, in addition to presenting a relatively smooth fracture surface. It can be observed that the films are constituted by a polymer matrix with a satisfactory degree of compaction, which is due to the influence of the two solvents. However, the film solubilized in THF (2d) has a more regular structure and fewer surface microcracks, which the more homogeneous drying process can explain, a characteristic common to THF-based processes.^28^

**Figure 2:**
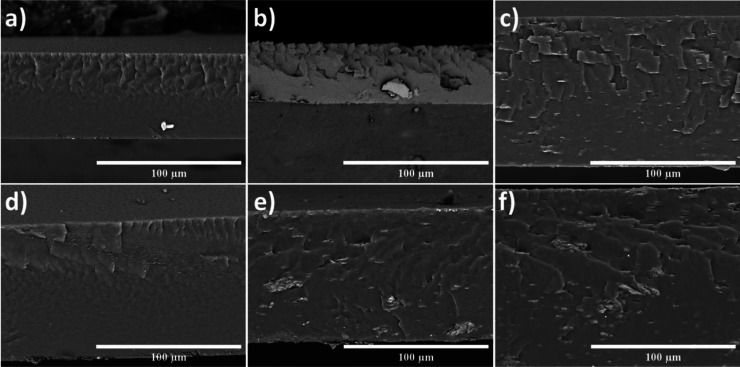
Cross-sectional SEM images of EVA-based films prepared by solvent evaporation: (a) ECHC0 %, (b) ECHC3 %, and (c) ECHC5 % (chloroform); and (d) ETC0 %, (e) ETC3 %, and (f) ETC5 % (tetrahydrofuran, THF).

In addition, the cross-sectional SEM images indicate that the films present a continuous thickness profile along the transverse direction, without abrupt variations or discontinuities. For a given composition, a good degree of thickness uniformity is observed, whereas a systematic increase in film thickness with increasing organoclay content can be inferred from the cross-sectional morphology. This behavior is attributed to the increase in solution viscosity induced by the presence of clay platelets during solvent evaporation.^16,21–23^

Nanocomposites synthesized with chloroform and clay additions at 3% and 5% exhibit distinct microstructures. The ECHC3 % film has micrometric agglomerates and empty regions along the cross-sectional profile near the top, forming an irregular zone. In the ECHC5 % film, an irregular appearance is observed in the upper region due to the overlap of microplates and low-definition interfaces. In contrast, a more uniform profile is observed in the lower region. These results agree with XRD, which shows the coexistence of peaks from the original clay and shifted peaks, suggesting a semicrystalline morphological state. According to Wilson et al.,^10^ this partially delaminated profile in nanocomposite structures tends to prevent the formation of ideal diffusion paths and promote the concentration of point stresses along the film, thus reducing mechanical and barrier performance, especially in regions with excess microvoids.

In nanocomposites produced with THF and clay concentrations of 3% and 5%, a slight change in texture is observed. The ETC3 % film has a fine grain size, with a considerable absence of micrometric agglomerates and microplates. The ETC5 % film maintains low-volume domains, but they are more elongated, with a preferred orientation parallel to the film plane.^30^ Small pull-outs are observed, as expected in fractures of fragile particulate systems. Still, the figures indicate a finer, continuous dispersion that is well integrated into the matrix, unlike that observed in samples solubilized with CHCl_3_.

The XRD and SEM results indicate that the final morphology of EVA/Cloisite 20A films is not governed solely by clay content but is strongly influenced by the solvent used during the evaporation process. While previous studies on EVA/clay nanocomposites primarily attribute structural variations to filler concentration and lamellar intercalation,^9,10^ the present results demonstrate that solvent selection significantly alters microstructural organization even at identical clay loadings. In particular, films prepared with chloroform exhibit more heterogeneous morphologies and lamellar agglomeration, whereas THF-based films show finer and more continuous dispersion, highlighting the decisive role of evaporation kinetics and solvent–polymer interactions in nanocomposite morphology.

Figure 3 shows the FTIR spectra of the polymer, clay, and composite films. The infrared spectra shown in Figure 3a show absorption behavior at the same wave numbers for both EVA and clay. For EVA, the peaks at 2915 and 2850 cm^–1^ correspond to the asymmetric and symmetric stretches of the –CH_2_– group.^28^ On the other hand, for organophilic clay, the absorptions at these positions in the spectrum result from the symmetric and asymmetric C–H stretching vibrations of the CH_3_ and CH_2_ groups present in the amine chains.^29^ The peaks located at 1460 and 1022 cm^–1^ in the EVA spectrum are related to the deformation of the –CH_2_– group and the absorption attributed to vinyl acetate, respectively. In clay, the absorption around 1460 cm^–1^ corresponds to the angular deformation of the CH_2_ group, and the peak at 1022 cm^–1^ results from the asymmetric stretching of Si–O.^28^

**Figure 3:**
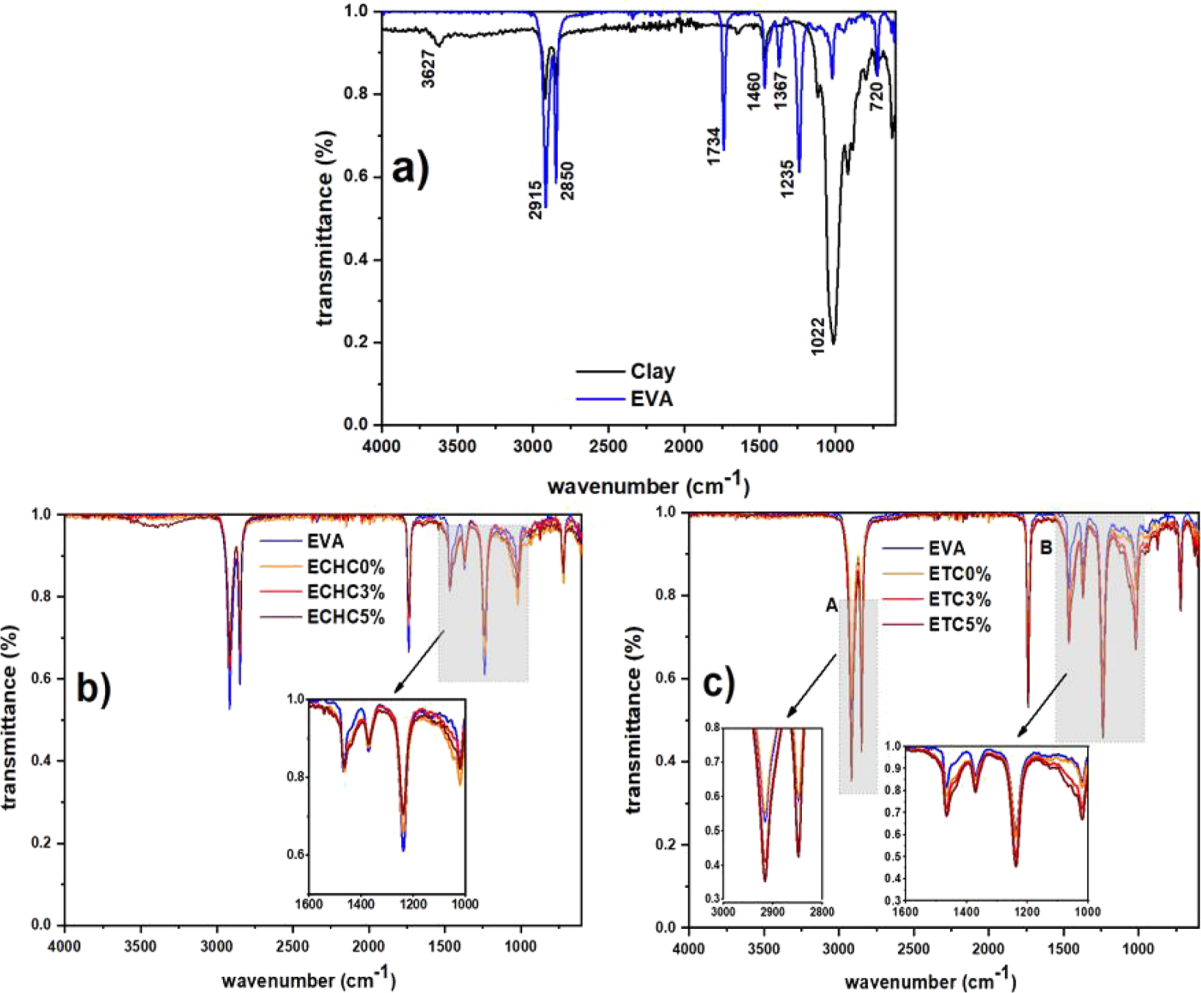
Infrared spectra of pure materials and nanocomposite films: (a) clay and EVA; (b) films produced with CHCl3; and (c) films produced with THF.

The spectra obtained for the composite film samples showed very similar behavior among themselves and closely matched the absorption pattern observed in the unreinforced polymer sample. This proves that there were no significant compositional changes. However, Figure 3b highlights a slight increase in absorption in the spectral region between 1000 and 1600 cm^–1^ corresponding to the angular deformation of –CH_2_– and the bond corresponding to silicate (Si–O) (highlight B). The other peaks that showed lower absorption intensity in the composite films than in the unreinforced EVA exhibit a systematic reduction, most easily observed at 2915, 2850, 1734, and 1235 cm^–1^. This may be due to the lower pressure of the sample on the ATR diamond.

The peaks between 1000 and 1600 cm^–1^ also showed higher intensities for the reinforced samples synthesized with THF, as shown in Figure 3c. In addition, Figure 3c shows higher peak intensities at 2915 and 2850 cm^–1^.

Figure 4 shows the thermogravimetric curves of the films solubilized with chloroform and tetrahydrofuran. EVA exhibits two stages of degradation. The first stage, occurring between 300 and 380 °C, corresponds to the degradation of acetic acid in vinyl acetate. The second degradation observed between 410 and 520 °C indicates the decomposition of ethylene chains.^30,31^ The TGA curve for the EVA sample showed a 6% mass loss up to 300 °C. On the other hand, the synthesized films (ETC and ECHC) showed greater thermal resistance up to 300 °C with a maximum mass loss of 2.9%. EVA then underwent rapid degradation, resulting in an abrupt mass loss of more than 19%. In contrast, ECHC films showed greater thermal resistance in this initial stage of degradation, as shown in Figure 4.

**Figure 4:**
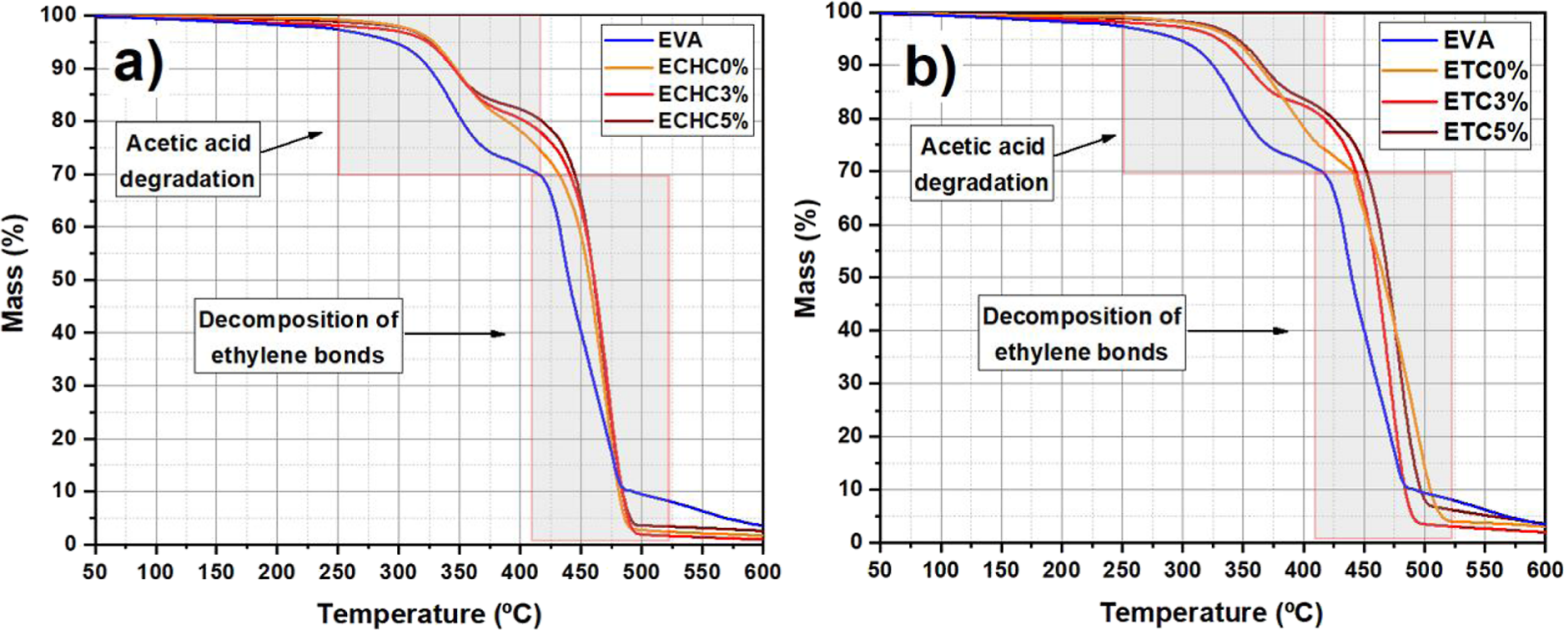
Thermogravimetric curves of films solubilized with (a) chloroform and (b) THF.

In the second stage of degradation, ECHC films showed greater resistance up to approximately 480 °C. However, the greater negative slope of the mass–loss curves for ECHC films resulted in greater degradation of the material when reaching 520 °C. The same behavior is observed in ETC films. Among the synthesized films, those with 5% reinforcement (ECHC5 % and ETC5 %) showed greater thermal resistance up to approximately 460 °C. Therefore, this clay reinforcement showed stability in the films and insignificant mass loss up to 300 °C, with less mass loss at higher temperatures.

According to Figure 4, a shift to higher temperatures is also observed at the beginning of the first degradation stage when clay is added to films solubilized with chloroform and THF (blue arrow). This can be explained by the diffusion effect, which hinders the emission of gaseous degradation products, thereby improving the material’s thermal stability.^30^

The differential scanning calorimetry (DSC) results shown in Figure 5 were obtained in the range of 30 to 150 °C. All DSC curves initially show a broad endothermic shoulder, on which the characteristic peaks are located between 50 and 100 °C. The peaks are around 54 and 86 °C and are attributed to the primary and secondary melting processes of EVA crystals.^32,33^ The low-temperature endothermic peak, *T*_low_ corresponds to the melting of smaller, imperfect crystallites. In contrast, the high-temperature endothermic peak *T*_high_ results from the melting of larger and more regularly formed crystallites that occur as a result of primary crystals being exposed to the open air.^34^ The reduction in the baseline observed in the curves for films with and without reinforcement indicates a decrease in the material’s thermal capacity, implying that less heat is required to achieve the same temperature variation.^35^ Figure 5 also shows the integrated areas representing the melting enthalpies, called Δ*H*_low_ and Δ*H*_high_.

**Figure 5:**
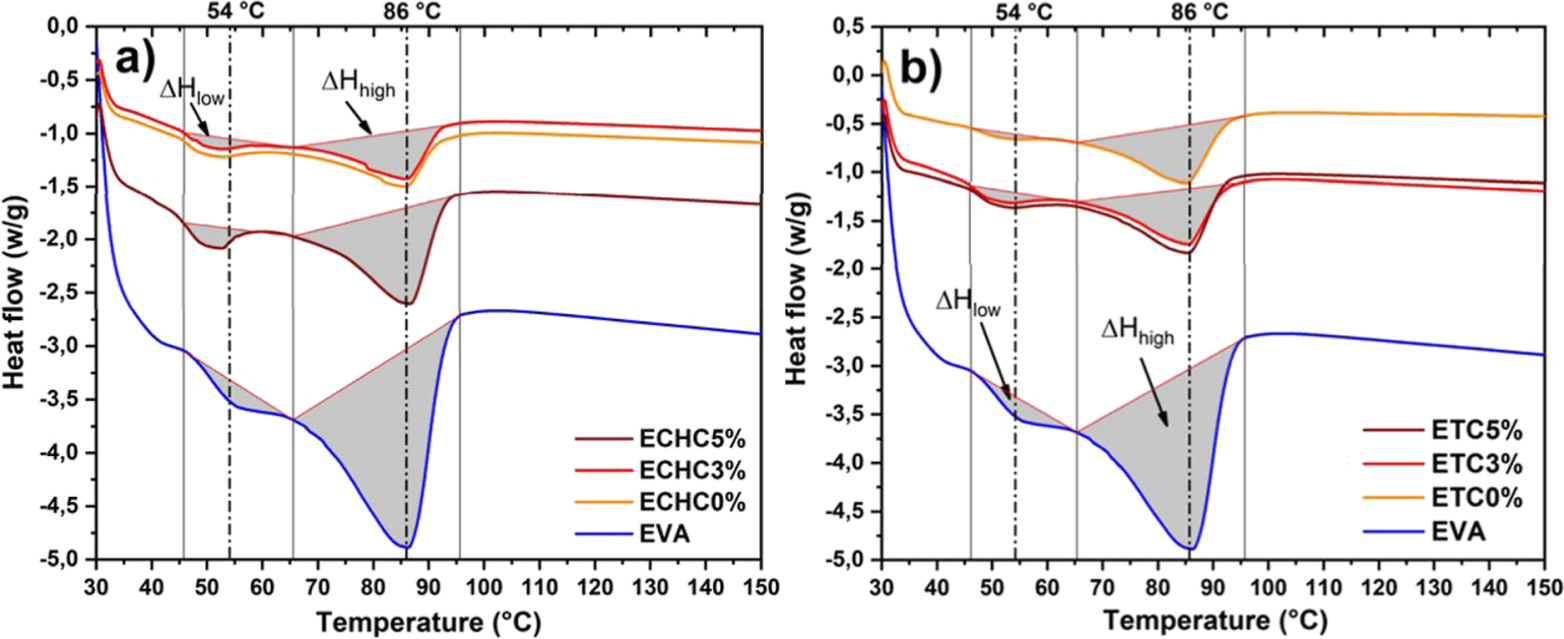
Differential scanning calorimetry of films solubilized with (a) chloroform and (b) THF.

The DSC results indicate that variations in the crystalline organization of EVA/Cloisite 20A films are not associated solely with the nucleating effect of the clay, which is widely discussed in the literature,^13,32^ but are also influenced by the solvent employed during the evaporation process. Films prepared with chloroform exhibit larger shifts in the low-temperature melting peak (*T*_low_), whereas those obtained with THF display more stable thermal responses, suggesting that evaporation kinetics and polymer chain mobility modulate the formation of crystalline domains. These findings demonstrate that the contribution of clay to EVA crystallinity should be interpreted synergistically with the solvent, rather than as an isolated effect.

Table 2 presents the melting temperatures and enthalpy values for the samples corresponding to the two peaks highlighted in Figure 5. The values obtained show that the films solubilized with chloroform (ECHC samples) exhibited a more pronounced deviation of the *T*_low_ melting peak to lower temperatures than the films solubilized with THF (ETC samples). The addition of clay as a reinforcement in the ECHC samples shifted the peak to slightly higher temperatures, indicating that the type of solubilizer contributed more significantly to the change in the films’ melting temperature than the reinforcement concentrations used in this approach. On the other hand, in the reinforced THF samples, this behavior could not be confirmed for the *T*_low_ temperature peak. The *T*_high_ value did not change as a function of the solubilizing agent and reinforcement, indicating that this higher melting temperature depends predominantly on the polymer matrix.

**Table 2:** Results of the Position of the Low (*T*_low_) and High (*T*_high_) Endothermic Peaks of Melting Temperature and their Respective Melting Enthalpies

films	*T*_low_ (°C)	Δ*H*_low_ (J/g)	*T*_high_ (°C)	Δ*H*_high_ (J/g)
EVA	54.0	1.91	86.0	28.34
ECHC0 %	50.3	1.02		5.39
ECHC3 %	50.9	1.03		5.77
ECHC5 %	51.4	7.34		12.11
ETC0 %	52.6	0.46		8.10
ETC3 %	51.8	1.29		7.83
ETC5 %	52.5	1.18		9.35

The solvent evaporation method hinders the formation of thick lamellar domains of ethylene, leading to a significant decrease in the enthalpy of fusion Δ*H*_high_ as shown in Table 2. The slightly higher Δ*H* values for films produced with 5% reinforcement indicate that clay causes the recovery of part of the crystallinity through nucleation. This behavior was more evident in the ECHC samples, which showed an increase in Δ*H*_low_ from 1.02 (without reinforcement) to 7.34 J/g e Δ*H*_high_ ranging from 5.77 (without reinforcement) to 12.11 J/g. It is worth noting that the reduction in melting enthalpy indicates an increase in amorphization and flexibility of the synthesized film structure.

The wettability test allowed us to verify the affinity of the films’ surfaces to distilled water. Measurements of contact angles in drop–surface interactions indicated that all samples exhibited hydrophilic behavior.^36^ Figure 6 shows the variation in contact angle measurements, obtained every 10 s until the end of the analysis. Both films produced with chloroform and tetrahydrofuran had contact angles below 90°. According to Ataeefard and Siamak (2011), the addition of clay to the polymer matrix can increase hydrophobicity by inducing pores that increase surface roughness.^37^ In fact, the average angle values increase with the addition of 3% clay as reinforcement in the films produced. However, the average angle returns to lower values with 5% reinforcement. The variation in the average contact angle value between the ETC0 % (76.48°) and ETC3 % (83.67°) samples, corresponding to 9.5%, is highlighted in Figure 6b.

**Figure 6:**
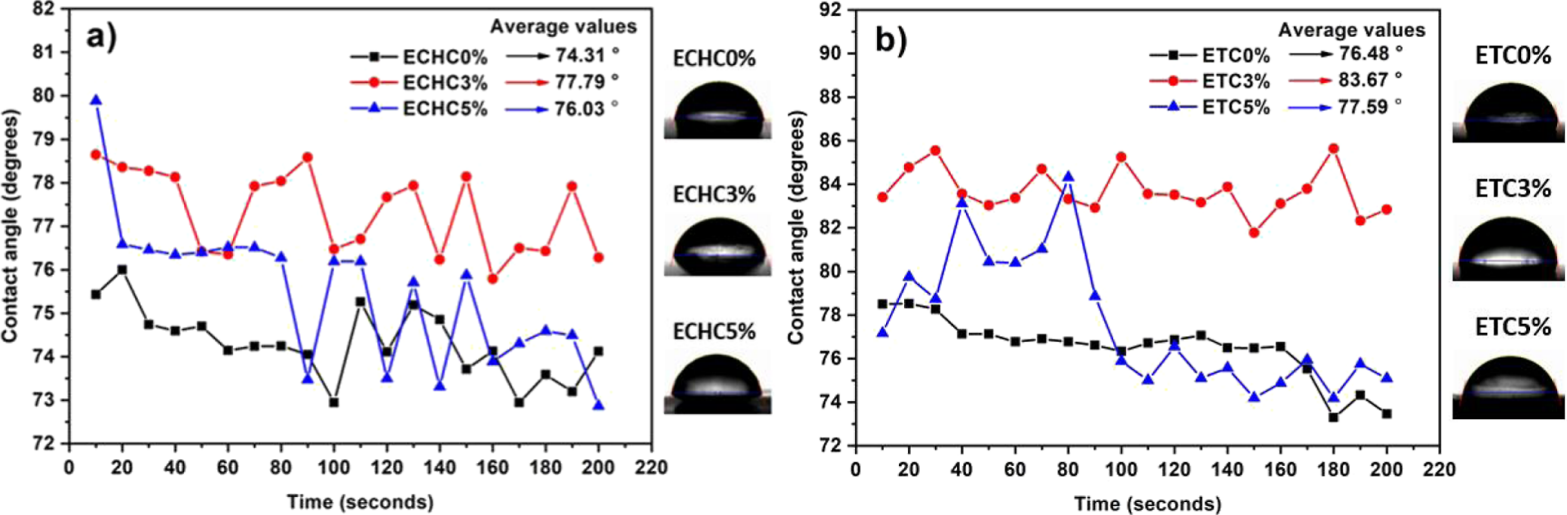
Variation of contact angle as a function of time for polymer films synthesized with (a) chloroform and (b) tetrahydrofuran.

The decrease in the average contact angle observed in samples with 5% reinforcement can be explained by possible saturation of the clay content, leading to pore filling. This saturation effect reduces microdefects and, consequently, porosity, thereby promoting greater surface mobility.

The effects of clay on the films produced can also be verified using the vapor transmission test. Figure 7 shows the variation in liquid mass with time due to the passage of water vapor through the polymer film, fit with a linear approximation. The EVA films produced with chloroform and tetrahydrofuran presented lines with smaller slopes as the clay reinforcement increased. This means that the increase in reinforcement reduced the films’ porosity, preventing the passage of vapor.

**Figure 7:**
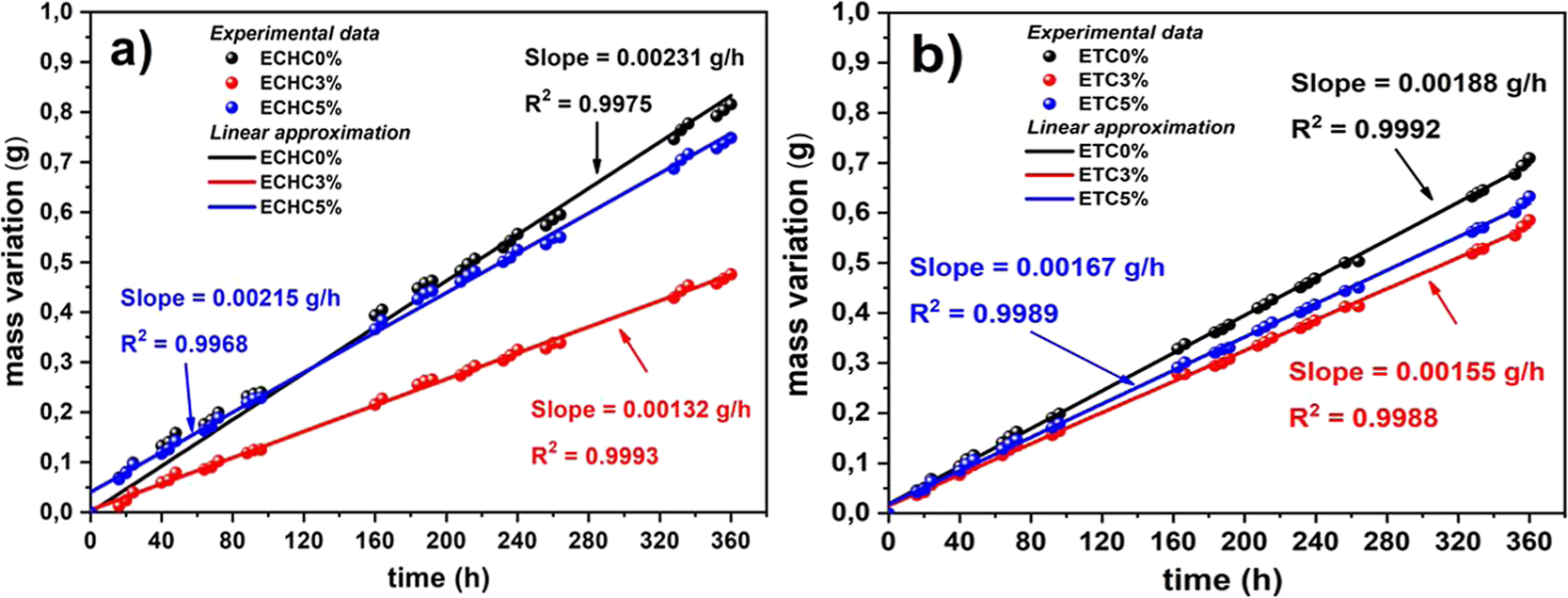
Vapor transmission through EVA polymer films produced with (a) chloroform and (b) tetrahydrofuran.

The contact angle and water vapor permeability results indicate that the surface and barrier properties of EVA/Cloisite 20A films are not governed solely by clay content but are also influenced by the solvent used during film preparation. Although the literature commonly attributes permeability reduction primarily to the increased tortuosity induced by clay lamellae,^10,11^ the results of this study show that films prepared with chloroform exhibit more pronounced reductions in WVT compared to those obtained with THF, even at identical filler loadings. This finding demonstrates that solvent-dependent microstructural organization plays a decisive role in the efficiency of the water vapor barrier.

Table 3 shows the water vapor transmission rate (WVT) of the produced films, which quantifies the amount of water vapor that passes through a film per unit time and area. This vapor transmission is directly related to the material’s microstructure, polarity, and crystallinity. The results show that adding 3% clay to the polymer film in chloroform reduced water vapor permeability by approximately 37%. However, adding 5% reinforcement increased permeability by 28% compared to the ECHC3% sample. A similar behavior occurs in films produced with THF. The addition of 3% clay reduced vapor permeability by approximately 15%, while 5% clay reduced it by 12% compared to the sample without reinforcement (ETC0 %).

**Table 3:** Water Vapor Transmission Rate (WVT) of Polymer Films

samples (%)	average WVT (g/(h.m^2^))	standard deviation
ECHC0	1.1390	0.2378
ECHC3	0.7166	0.0245
ECHC5	0.9167	0.28
ETC0	1.0556	0.0373
ETC3	0.8936	0.0572
ETC5	0.9244	0.0765

Figure 8 shows the mechanical behavior of the films subjected to tensile testing up to 400% elongation. The films produced with chloroform did not break, exhibiting excessive plasticity, a common characteristic of products based on EVA, a highly flexible thermoplastic. The addition of clay increased the films’ stiffness, requiring greater tension for the same deformation than the unreinforced sample (ECHC0 %). On the other hand, the films produced with THF showed lower plasticity. The ETC0 % and ETC3 % films break at around 150% elongation. However, the 3% reinforcement increased stiffness, as shown in Figure 8b. The ETC5% sample showed greater stiffness and plasticity, without breaking during the test.

**Figure 8:**
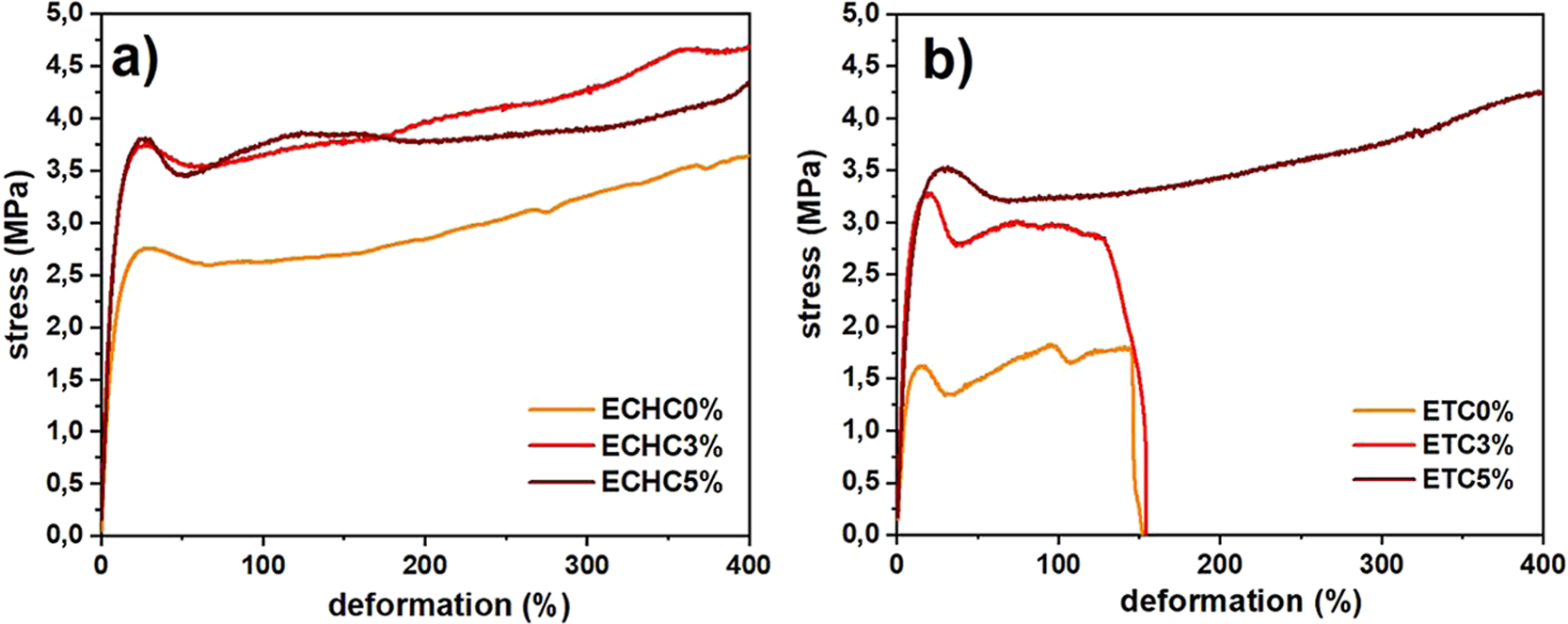
Stress–strain curves obtained in tensile tests of polymer films (a) ECHC and (b) ETC.

The increase in stiffness is quantified in the tensile test by Young’s modulus, as shown in Table 4. Both films produced with chloroform and those produced with THF showed that incorporating clay resulted in a significant increase in stiffness compared to the pure polymer film. The films with chloroform and 3% and 5% reinforcement showed increases of approximately 43% and 33% in Young’s modulus compared to the ECHC0 % sample. In films produced with THF, the reinforcement had a more pronounced effect. The ETC3 % sample showed approximately twice the stiffness of the ETC0 % sample, while the ETC5 % sample showed a 68% increase due to the addition of clay. The reduction in Young’s modulus of films with 5% clay compared to films with 3% can be explained by less efficient dispersion and the consequent formation of possible agglomerates at higher reinforcement contents.

**Table 4:** Young’s Modulus of ECHC and ETC Polymer Films with Different Clay Contents (0, 3, and 5% by Mass)

samples (%)	Young’s modulus (MPa)
ECHC0	30.56
ECHC3	43.83
ECHC5	40.67
ETC0	22.36
ETC3	46.44
ETC5	37.74

The results indicate that a clay content of 3 wt % represents a near-optimal condition for the systems investigated, in which the reinforcing effects are maximized without significant loss of dispersion quality. At higher loadings (5 wt %), the reduced mechanical gains can be attributed to agglomerate formation and decreased stress transfer efficiency, in agreement with the behavior widely reported for polymer/clay nanocomposites in the literature.^9,10,13^

## Conclusion

This study examined the effects of solvent choice (chloroform and THF) on the production of EVA films by solvent evaporation, as well as the influence of low organoclay contents (3% and 5%) as reinforcement. The results demonstrate that the structural and functional properties of EVA/Cloisite 20A films are governed by the combined effect of solvent selection and clay loading, rather than by clay content alone.

Chemical, structural, and morphological analyses (XRD, FTIR, and SEM) showed that the solvent evaporation process promotes clay dispersion within the polymer matrix, leading to distinct microstructural organizations depending on the solvent employed. Films prepared with chloroform exhibited a more heterogeneous morphology across the film thickness, whereas THF-based films showed finer and more continuous lamellar dispersion. These differences directly influenced the thermal, mechanical, and barrier responses of the nanocomposites.

The clay-reinforced films exhibited improved thermal stability, with reduced mass loss up to approximately 300 °C. The increase in low-temperature melting enthalpy (Δ*H*_low_) for reinforced samples indicates changes in crystalline organization, which contributed to enhanced material stiffness. Mechanical testing revealed a significant increase in Young’s modulus, particularly for the ETC3 % sample, which exhibited an increase of over 100% compared to the unreinforced ETC0 % film. However, at higher clay content (5%), the mechanical gains were less pronounced, suggesting the onset of less efficient stress transfer due to microstructural heterogeneity.

Barrier and wettability analyses showed that clay incorporation increased the contact angle and reduced water vapor transmission, with the ECHC3 % film presenting a permeability reduction of approximately 37%, while the ETC3% film showed a reduction of about 15% relative to their respective neat films. These results highlight that an intermediate clay content (3%) provides an optimal balance between dispersion efficiency and functional performance.

The findings demonstrate that solvent selection acts as an active processing parameter that modulates structure–property relationships in EVA/organoclay nanocomposite films. In particular, the combination of chloroform and moderate clay loading proved effective in enhancing thermal, mechanical, and barrier properties, reinforcing the relevance of processing conditions in the rational design of EVA-based functional films.
